# Scanning Electron Microscopy of Antennae and Mouthparts of *Mezira yunnana* Hsiao (Hemiptera: Aradidae): Specialized Microstructures Reflecting Adaptation to Mycetophagy

**DOI:** 10.3390/insects14040333

**Published:** 2023-03-29

**Authors:** Shiyu Zha, Zhiyao Wang, Li Tian, Yisheng Zhao, Xiaoshuan Bai, Zhaoyang Chen, Wanzhi Cai, Xinyu Li, Hu Li

**Affiliations:** 1 Department of Entomology and MOA Key Lab of Pest Monitoring and Green Management, College of Plant Protection, China Agricultural University, Beijing 100193, China; 2 College of Life Science and Technology, Inner Mongolia Normal University, Hohhot 010022, China; 3 College of Forestry, Beijing Forestry University, Beijing 100083, China

**Keywords:** Aradidae, antennae, mouthparts, sensilla, microstructures, feeding habits

## Abstract

**Simple Summary:**

Antennae and mouthparts are key organs for food searching and feeding in insects. In the present study, the microstructures of the antennae and mouthparts of the flat bugs *Mezira yunnana* Hsiao were demonstrated. Five types of antennal sensilla and three types of labial sensilla were documented. Labial tip gustatory sensilla form a small group of only three pairs, which is essentially different in this species from other true bugs. The labial tip is constricted distally, which is rarely observed in other pentatomomorphans. Teeth on the external surface of the mandibular apex are ridge-like and uniform. These characteristics may be associated with the species’ unique fungi-feeding habit.

**Abstract:**

Many species of the family Aradidae (also known as flat bugs) feed on fungal mycelia and fruiting bodies. In order to better understand the morphological adaptation to this unique feeding habit, we examined the microstructure of antennae and mouthparts of an aradid species, *Mezira yunnana* Hsiao, using scanning electron microscope, and documented the fungal feeding process under laboratory conditions. The antennal sensilla include three subtypes of sensilla trichodea, three subtypes of sensilla basiconica, two subtypes of sensilla chaetica, sensilla campaniformia, and sensilla styloconica. The apex of the second segment of flagellum has a large number of various sensilla forming a sensilla cluster. The labial tip is distally constricted, which is rarely observed in other Pentatomomorpha species. The labial sensilla include three subtypes of sensilla trichodea, three subtypes of sensilla basiconica, and a sensilla campaniformia. The tip of the labium has only three pairs of sensilla basiconica III and small comb-shaped cuticular processes. The external surface of the mandibular apex has 8–10 ridge-like central teeth. A series of key morphological structures associated with mycetophagous feeding habit were identified, which will facilitate future studies on adaptive evolution of species in Pentatomomorpha as well as in other heteropteran lineages.

## 1. Introduction

Heteroptera (true bugs) are the most diverse group in the order Hemiptera, which is widely distributed around the world, occupying various types of habitats and exhibiting diverse feeding habits (phytophagy, carnivore, hematophagy, mycetophagy (fungal feeding), and coprophagy) [1,2,3,4]. Adaptive changes in feeding-related morphology have been hypothesized to be a key driver of species diversification in this group [5]. During their evolutionary history, the antennae and mouthparts of Heteropteran species have modified extensively to serve their unique functions [1,4,5,6,7]. The various types of sensory structures distributed on the antennae of true bugs form a sensilla system, which is the center for receiving signals, including the chemical stimuli during long-distance orientation, and mechanical stimuli in contact with the surface of hosts and mates. Thus, the antennae play an important role in the host-recognition, mating, oviposition, defense, and other behaviors during the life history of true bugs [6,8,9,10]. The labial tip, which contacts the host surface during the host selection and feeding process, is usually covered with numerous sensilla functioning as chemoreceptors or mechanoreceptors [5,7,11,12,13,14]. The labium houses the stylet fascicle, which penetrates the food tissue during feeding. The morphology of the stylets adapted to the specialized food sources largely varies among species with different feeding habits [13,15].

To date, the morphology and microstructures of the antennae and mouthparts have been characterized in many heteropteran species with various feeding habits, including phytophagous, predatory, and blood-feeding habits [8,16,17,18]. In contrast, research on mycetophagous species is rare. In Heteroptera, mycetophagy is mainly recorded in the family Aradidae (commonly called the “flat bugs”) [12,19,20]. Aradidae is a group with 8 subfamilies and more than 2000 species in the world [21,22]. Aradidae are mostly flattened in form and somber in color, usually live under the bark of dead tree or in leaf litter [23,24]. Many species of this family have been reported to be mycetophagous, feeding on mycelium and fruiting bodies, thus some species are even considered agricultural pests, such as *Mezira poriaicola* Liu and *Mezira membranacea* Fabricius [25,26]. The stylets of the flat bugs are extremely elongated, equivalent to body length or more, which were believed to be an adaptation to its mycetophagous feeding habits [12,20,24,27].

**Figure 1:**
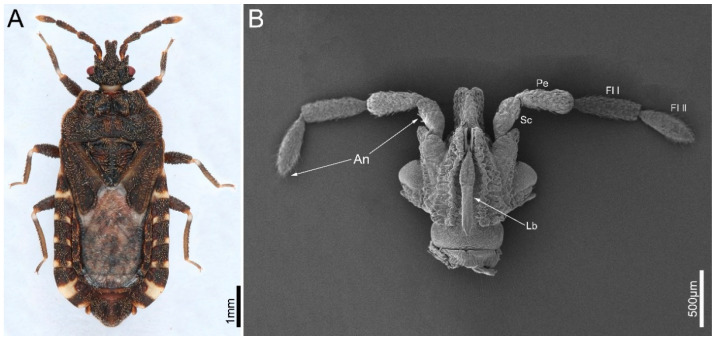
Habitus and general head morphology of *Mezira yunnana*. (**A**) Dorsal habitus of the male; (**B**) morphology of head with antennae and labium. An: antennae; Fl I-II: flagellum I-II; Lb: labium; Pe: pedicel; Sc: scape.

In present study, we focus on *Mezira yunnana* Hsiao, 1964 (Figure 1A), an aradid species distributed in southern China. We characterize microstructures of the antennae and mouthparts using scanning electron microscope (SEM). We perform morphological comparisons with other pentatomomorphan species to determine key morphological specializations to fungal feeding. Then, we describe its fungal feeding behavior to demonstrate how some specialized feeding structures of this species function to facilitate fungal feeding.

## 2. Materials and Methods

### 2.1. Insects Collecting

Forty adults of *M. yunnana* were captured in Changpoling National Park, Guizhou Province. Colonies were established in the laboratory at China Agricultural University, Beijing, China.

### 2.2. Samples for SEM

Adult males (*n* = 7) and females (*n* = 6) were cleaned three times while using an ultrasonic cleaner (KQ2200E, Kunshan Ultrasonic Instrument Co, Ltd.), 30 s each time. Dehydration used serial baths of 85%, 90%, and 95% each for 15 min, and 100% ethanol twice each for 10 min. The materials were air dried, coated with a film of gold (EIKO IB-2), and then imaged with a scanning electron microscope HITACHI S-3400N at 10 kV in the scanning microscopy laboratories at the College of Biological Science of China Agricultural University.

### 2.3. Feeding Behavior

Adults were reared in one plastic container 36 cm long, 27 cm wide, and 21 cm high, under a largely constant temperature of 26 ± 0.5 °C and RH of 70 ± 10%. They were provided with the mycelium and fruiting bodies of *Pleurotus ostreatus* Kummer and *P. citrinopileatus*. Images of the feeding processes were taken using a Canon EOS 7D.

### 2.4. Image Processing and Terminology

Photographs and SEMs were observed and measured after being imported into Adobe Photoshop 2021 (Adobe Systems, San Jose, CA, USA). The sensilla were classified according to their external morphology, distribution, and position. For classification of sensilla, the systems of Altner and Loftus [28] were used in addition to the more specialized nomenclature from other studies [5,20,29].

## 3. Results

### 3.1. Antennae

**Figure 2:**
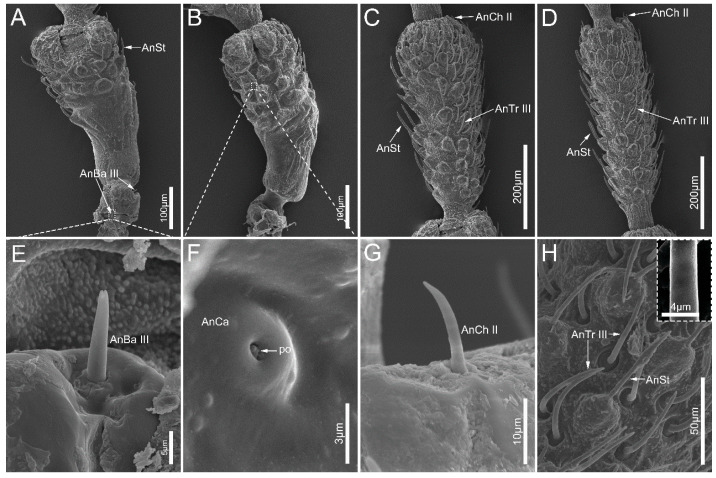
Scape, pedicel, and the first segment of flagellum of *Mezira yunnana*. (**A**) View of scape showing the localization of AnBa III; (**B**) view of scape showing the localization of AnCa; (**C**) lateral view of pedicel; (**D**) lateral view of the first segment of flagellum; (**E**) AnBa III on scape; (**F**) AnCa on pedicel; (**G**) AnCh II on the first segment of flagellum; (**H**) AnSt and AnTr III on the first segment of flagellum, with porous wall of antr III shown in the box; AnBa III, antennal sensilla basiconica III; AnCh II, antennal sensilla chaetica II; AnCh III, antennal sensilla chaetica III; AnCa, antennal sensilla campaniformia; AnSt, antennal sensilla styloconica; AnTr III, antennal sensilla trichodea III; po, pore.

**Figure 3:**
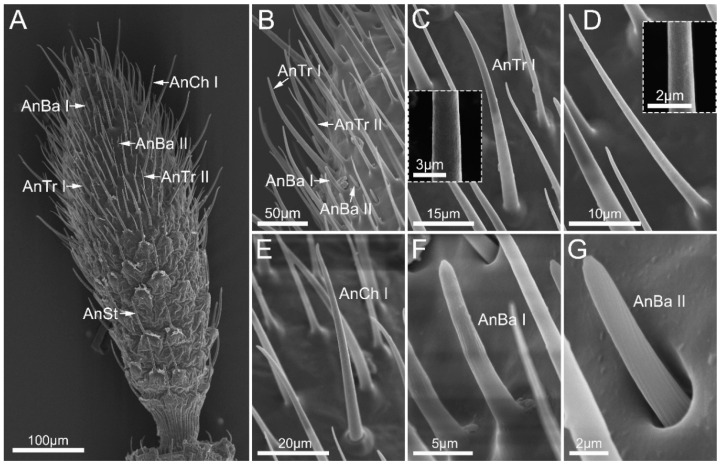
The second segment of flagellum of *Mezira yunnana*. (**A**) View of the second segment of flagellum; (**B**) enlarged view showing the distribution of the sensilla on the second segment of flagellum. (**C**) antennal sensilla trichodea I (AnTr I), with porous wall shown in the box; (**D**) antennal sensilla trichodea II (AnTr II), with porous wall shown in the box; (**E**) antennal sensilla chaetica I (AnCh I); (**F**) antennal sensilla basiconica I (AnBa I); (**G**) antennal sensilla basiconica II (AnBa II).

The antennae of *M. yunnana* comprise four segments (Figure 1B). The first (scape, Sc) and second (pedicel, Pe) segment is stick-shaped, while the third segment (the first segment of flagellum, Fl I) is cylindrical, and the fourth segment (the second segment of flagellum, Fl II) is shuttle-shaped. The base of scape is bending (Figure 2A,B). These segments are mostly covered with small nodes, but relatively smooth at the apical 2/3 of the Fl II and basal 1/3 of the Sc (Figure 3A), which is covered with denser sensilla to form a sensilla cluster. Five types of sensilla were found on the antennae, namely sensilla trichodea, sensilla basiconica, sensilla chaetica, sensilla campaniformia, and sensilla styloconica. The length and basal diameter of each antennal segment was measured (Table 1).

Antennal sensilla trichodea (AnTr) are hair-shaped sensilla with inflexible sockets and porous walls. Based on the length and shape, we can distinguish three subtypes of AnTr, including antennal sensilla trichodea I (AnTr I), which are straight but have a tapered tip (Figure 3C); antennal sensilla trichodea II (AnTr II), which are slender, straight and shorter than AnTr I (Table 2, Figure 3D) and distributed on the apex of the last antennal segment; and antennal sensilla trichodea III (AnTr III), which are curved (Figure 2H) and found on the dorsal surface of antennal segments.

We distinguished three subtypes of antennal sensilla basiconica (AnBa), including antennal sensilla basiconica I, II, and III (AnBa I, AnBa II, and AnBa III). AnBa I and AnBa II are peg-shaped sensilla with blunt tips and straight longitudinal grooves exceeding half length of the upper part of the sensilla. AnBa I are longer than AnBa II, and both of them are inserted in inflexible sockets (Table 2, Figure 3F,G). The base of antennal sensilla basiconica II is hidden in a shallow and open cavity. Both AnBa I and AnBa II are found only on the Fl II. AnBa III are longer than AnBa II and possesses smooth walls and flexible sockets, and were only found on Sc (Table 2, Figure 2E).

Antennal sensilla chaetica (AnCh) have thick walls and narrow tips, and they are inserted in flexible sockets. Two subtypes of AnCh can be distinguished. Antennal sensilla chaetica I (AnCh I) are long and straight, which only distribute on the apex of Fl II (Figure 3E). Antennal sensilla chaetica II (AnCh II) are significantly shorter than AnCh I, and they were found on all antennal segments expect Fl II near the junction between segments (Figure 2D,H).

Antennal sensilla campaniformia (AnCa) have a disc-like shape with a pore (po) on the convex surface, and were found on the scape (Figure 2B,F).

Antennal sensilla styloconica (AnSt) have narrow tips and are carried by a cylindrical basal socket of the cuticle, evidently projected over the surface. This type is distributed on all antennal segments with higher density on the dorsal surface than on the ventral surface (Figure 2A–D,H and Figure 3A).

### 3.2. Mouthparts

#### 3.2.1. Gross Morphology of Mouthparts

**Figure 4:**
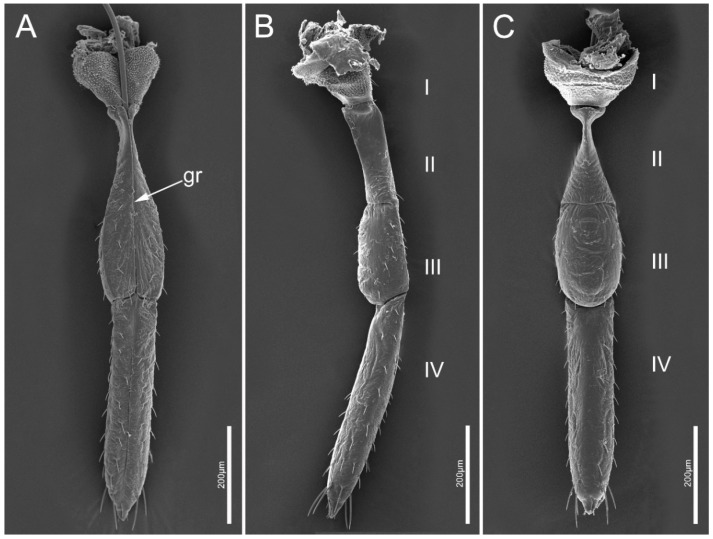
General morphology of the labium of *Mezira yunnana*. (**A**) Ventral view showing the longitudinal groove (gr); (**B**) lateral view showing the four labial segments (I–IV); (**C**) dorsal view showing the four labial segments (I–IV).

The mouthparts of *M. yunnana* arise from the anterior part of the head capsule and extend back along the ventral side of the body, consisting of tubular four-segmented labium (Lb) and a stylet fascicle comprising two maxillary stylets (Mx) and two mandibular stylets (Md). The labium has a longitudinal groove (gr) located in the middle of the dorsal side, surrounding the stylet fascicle (Figure 4A). The labrum (Lm) of *M. yunnana* is vestigial and invisible. The length of the stylet fascicle was measured (Table 1).

#### 3.2.2. Labium

Various types of sensilla are symmetrically distributed on all labial segments on each side of the labial groove. The four labial segments display marked difference in size and morphology.

**Figure 5:**
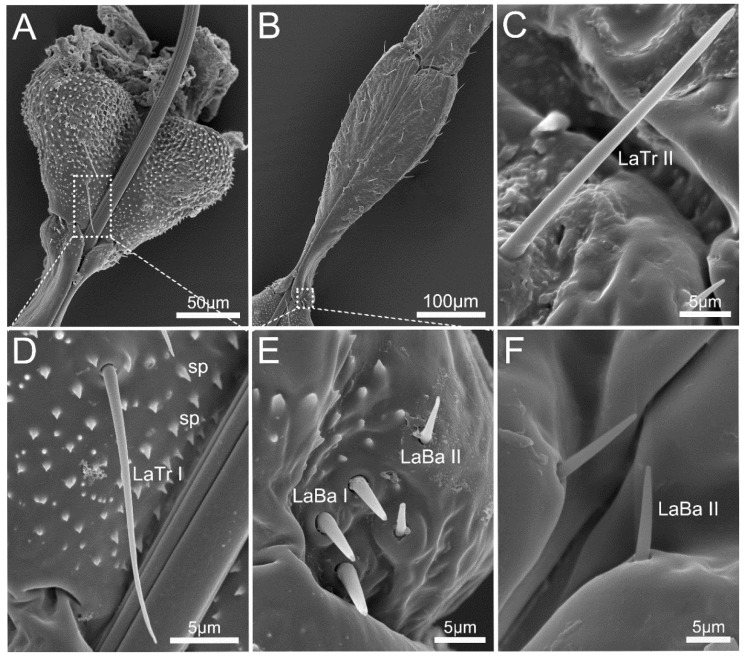
Labium of *Mezira yunnana*. (**A**) View of the first labial segment; (**B**) view of the second and third labial segments; (**C**) labial sensilla trichodea II (LaTr II); (**D**) labial sensilla trichodea I (LaTr I) and small spine-shaped projections (sp); (**E**) labial sensilla basiconica I (LaBa I) and labial sensilla basiconica II (LaBa II); (**F**) LaBa II near the junction.

The base of the first segment is broad but abruptly narrowed at the end (Figure 5B). The ventral surface of the first segment is covered with numerous small spine-shaped projections (sp), with hair-shaped labial sensilla trichodea I (LaTr I), sparsely arranged (Figure 5D).

Small spine-shaped projections (sp) covered the junction of the first and second segment, which has three pairs of labial sensilla basiconica I (LaBa I) and two pairs of labial sensilla basiconica II (LaBa II) point to base (Figure 5E). LaBa I has a blunt tip and flexible socket, and are broader and longer than LaBa II (Table 2). The base of the second segment is intumescent, followed by a sharp constriction, then gradually widens to the end. The second segment is partly reduced by fusing with the third segment ventrally. The third segment is slightly tapering toward the tip. Another pair of LaBa II is arranged at the end of the third segment (Figure 5F). Labial sensilla trichodea II (LaTr II) are also hair-shaped but longer than LaTr I, and distributed sparsely on the second and third segment (Table 2, Figure 5C).

**Figure 6:**
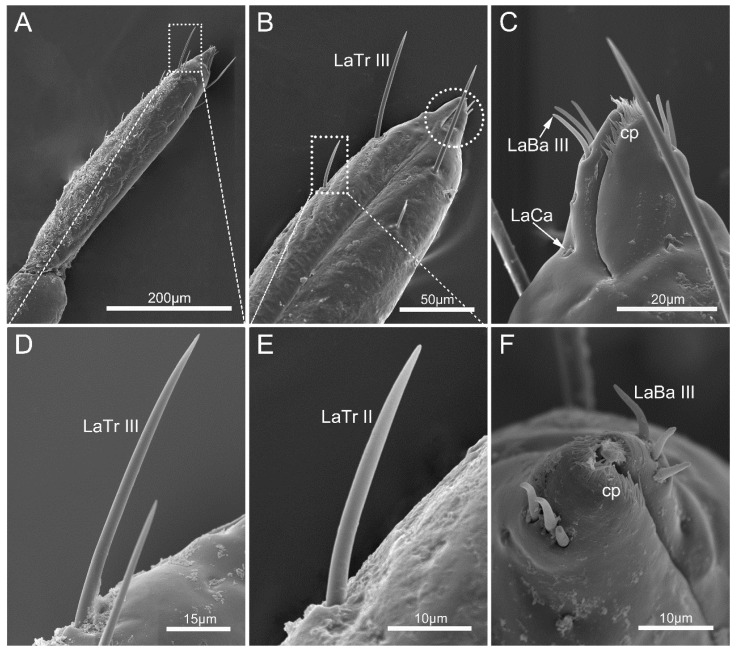
The fourth labial segment of *Mezira yunnana*. (**A**) General view of the fourth labial segment; (**B**) enlarged view showing labial sensilla trichodea III and labial sensilla II, with significantly narrowed labial tip shown in the circle; (**C**) enlarged view showing cuticular processes (cp), labial sensilla basiconica III (LaBa III), and labial sensilla campaniformia (LaCa); (**D**) labial sensilla trichodea III (LaTr III); (**E**) labial sensilla trichodea II (LaTr II); (**F**) vertical view of labial tip.

The fourth labial segment is the longest. It is cone-shaped and has a sharp contraction at the end, forming a significantly narrowed labial tip (Figure 6A,B). A large number of LaTr II are arranged on each side of the labial groove and the dorsal surface (Figure 6E). Slightly curved labial sensilla trichodea III (LaTr III) are arranged in the area near the apex (the labial subapex), which is the longest labial sensilla trichodea (Table 2, Figure 6D).

The labial tip is cleft and distinctly divided into two asymmetrical lateral lobes, and the left lobe is slightly wrapped around the right on the ventral surface (Figure 6C,F). Three pairs of labial sensilla basiconica III (LaBa III) encircle the labial tip, which is slightly curved (Figure 6C,F). Several labial sensilla campaniformia (LaCa) are located on the surface near the LaBa III, each of which is leaf-shaped (Figure 6C). Small comb-shaped cuticular processes (cp) densely encircle the opening (Figure 6C,F).

#### 3.2.3. Stylet Fascicle

**Figure 7:**
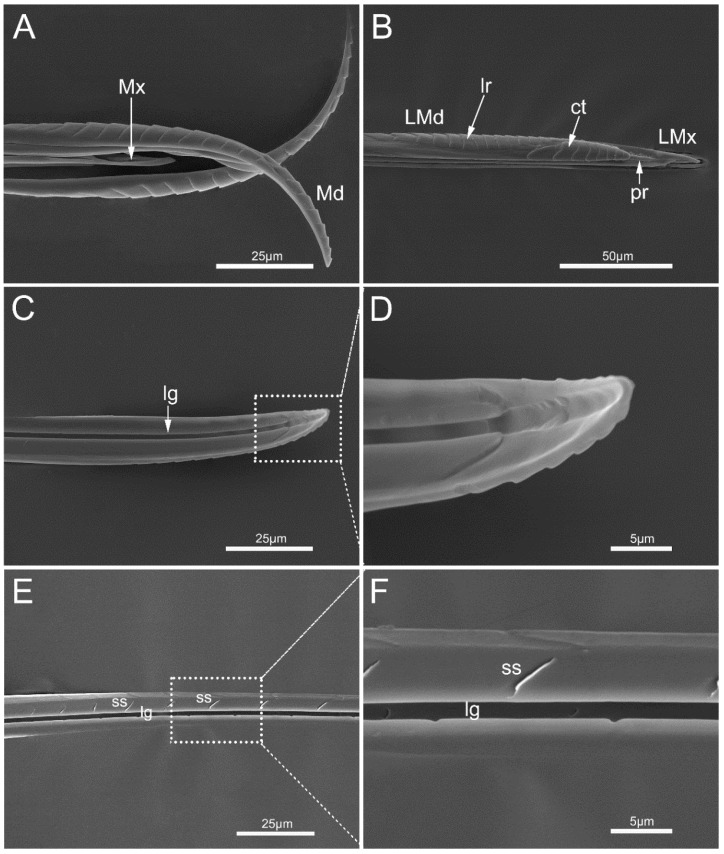
Mandibular stylets of *Mezira yunnana*. (**A**) Mandibular stylets (Md) and maxillary stylets (Mx); (**B**) left mandibular stylet (LMd) and left maxillary stylet (LMx) with an external longitudinal process (pr); (**C**) interior view of mandibular stylet showing longitudinal groove (lg); (**D**) enlarged view of the inner surface of mandibular apex; (**E**) interior view of mandibular stylet showing longitudinal groove and small spikes (ss); (**F**) enlarged view of the small spikes; ct, central teeth; lr, lateral ridges.

Stylet fascicle of *M. yunnana* is very long, about 1.5 times as long as the body (Table 1). Two separated mandibular stylets (Md) and two interlocked maxillary stylets (Mx) compose the long stylet fascicle (Figure 7A). Maxillary stylets are slightly longer than the mandibular stylets.

The left mandibular stylet (LMd) and right mandibular stylet (RMd) are concave, internally forming a groove to enclose the maxillary stylets. Eight to ten central teeth (ct) are ridge-like and are present on the external surface of the mandibular stylet at the apex along with a row of lateral ridges (lr) (Figure 7B). The inner surface of the mandibular stylet is relatively smoother, with a deep longitudinal groove (lg) on it (Figure 7C,E,F). Some small spikes (ss) are longitudinally arranged on the middle of the inner surface (Figure 7E,F).

**Figure 8:**
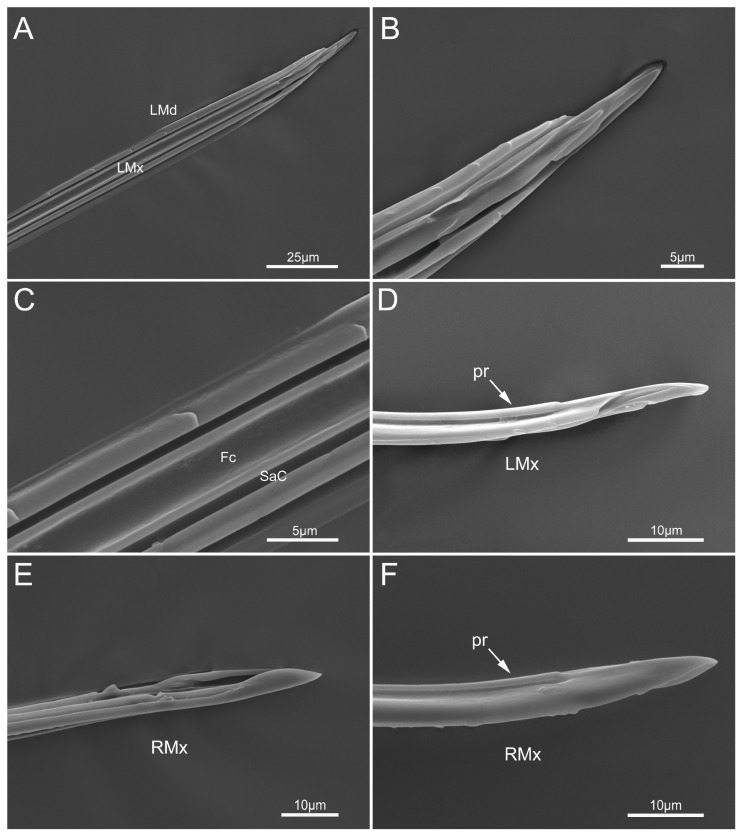
Maxillary stylets of *Mezira yunnana*. (**A**) Left mandibular stylet (LMd) and left maxillary stylet (LMx); (**B**) enlarged view of the apex; (**C**) enlarged view showing the food canal (Fc) and salivary canal (SaC); (**D**) external view of left maxillary stylet showing the external longitudinal process (pr); (**E**) apex of right maxillary stylet (RMx); (**F**) external view of right maxillae (RMx) showing the external longitudinal process.

The external and inner surfaces of the two asymmetrical maxillary stylets are both smooth, but equipped with an external longitudinal process (pr) that engages the grooves of the mandibular stylets (Figure 8A–F). The maxillary stylets have a narrowed and blunt apex, and the apex of the left maxillary (LMx) is narrower than the right maxillary (RMx). The right maxillary stylet and left maxillary stylet (LMx) form a food canal (Fc) and a salivary canal (SaC), and the central food canal is much wider in diameter than the salivary canal (Figure 8C).

### 3.3. Feeding Processes by M. yunnana

The feeding process involves seven steps: probing, orienting by antennae, orienting by rostrum, inserting, sucking, withdrawing, and resetting.

**Figure 9:**
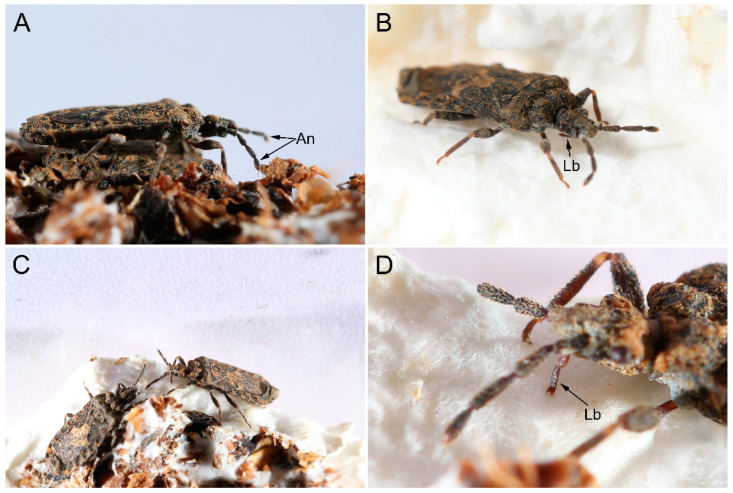
Feeding process of *Mezira yunnana*. (**A**) Swaying antennae; (**B**) orienting by rostrum; (**C**) feeding on a fruiting body; (**D**) enlarged view showing the position of the labial tip. An, antennae; Lb, labium.

Before finding the host, *M. yunnana* will sway antennae while walking (Figure 9A). The second flagellum reclinates and touches the ground frequently. When *M. yunnana* find the mycelium, they rotate their rostrum to touch the host, further orienting a suitable position on the host surface, generally an area where mycelium grows vigorously and forms a thick colony (Figure 9B). After selecting a suitable position to feed, *M. yunnana* inserts the labial tip into the mycelium (Figure 9C,D). The angle of the rostrum to the head is about 90°. Whereafter, the long, flexible stylet fascicle inserts into the host and initiates sucking. By constantly adjusting the position of stylet fascicle in the host, they can stay in one suitable area for a long time to feed. When the feeding is over, *M. yunnana* moves its body to pull out the stylet fascicle from the host. Finally, the rostrum rotates into the rostrum groove beneath on the ventral surface of the head. A similar behavioral process was also found during fruiting-body feeding.

## 4. Discussion

### 4.1. Specialized Arrangement of Antennal Sensilla in Flat Bugs

The types of antennae sensilla are associated with signal perception, which is important for insects that need to respond to complex environmental factors [30,31,32,33]. In this study, three subtypes of antennal sensilla trichodea (AnTr I-III), three subtypes of antennal sensilla basiconica (AnBa I-III), two subtypes of antennal sensilla chaetica (AnCh I,II), an antennal sensilla campaniformia (AnCa), and an antennal sensilla styloconica (AnSt) of *M. yunnana* were identified based on morphological characteristics. Combined with previously published studies [5,17,28,34,35], their potential functions were hypothesized. Antennal sensilla with grooved or porous walls and inflexible sockets are suggested to be chemoreceptory (i.e., AnBa I and AnBa II). Antennal sensilla that carry out mechanoreceptive function generally have flexible sockets (i.e., AnCh I). Mechanoreceptors located at the joint between segments and/or the cuticle areas subjected to stress are considered to play the role of proprioception (i.e., AnBa III, AnCh II). In previous research, antennal sensilla styloconica have been shown to function in thermos-hygroreception [28]. The types of antennal sensilla in Aradidae are summarized (Table 3). The most notable character of the antennae of the mycetophagous flat bugs is that the apex of the second segment of flagellum has large number of different types of sensilla, forming a sensilla cluster [19]. Flat bugs generally feed on fungi that grows in relatively hidden and less heterogeneous microhabitats, such as under the bark of dead trees and/or in leaf litter [23,24]. We suggest that the specialized arrangement of antennae sensilla of flat bugs is associated with their unique habit. Further studies are necessary to test this inference.

### 4.2. Unique Morphology of Mouthparts Adapted to Fungal Diet

The morphology of the labrum varies depending on feeding habits and mechanisms [1]. Observations of the mouthparts of *Erthesina fullo* Thunberg had many wrinkles on the ventral surface of the labrum, which may add flexibility to the labrum, allowing deeper stylet penetration [14]. *E. fullo* mainly feed on tree sap, thus the labrum structure may facilitate penetrating into tough plant surfaces, such as tree bark. The primary food type consumed by *M. yunnana* is the mycelium or fruiting body with a relatively soft surface texture that easy to penetrate, which may explain the absent of labrum in flat bugs. In this study, three subtypes of labial sensilla trichodea (LaTr I-III), three subtypes of labial sensilla basiconica (LaBa I-III), and a labial sensilla campaniformia (LaCa) of *M. yunnana* were distinguished based on morphological characteristics. Combined with previous studies [5,13,14,29,36,37,38,39,40], their potential functions are summarized (Table 2). Sensilla basiconica on the labial tips are hypothesized to carry out a contact-chemoreceptive function [5,13,14,16,29]. Interestingly, only three pairs of LaBa III near the opening at the labial apex, which is much less compared to other previously examined phytophagous or predaceous pentatomomorphans. For example, at least seven pairs of gustatory sensilla were found on the labial tip in *Halyomorpha halys* (*Stål*) (Pentatomidae; phytophagy) [5], eight in *Perillus bioculatus* (Fabricius) (Pentatomidae; predaceous) [16], twelve in *Pyrrhocoris sibiricus* Kuschakevich (Pyrrhocoridae; phytophagous) [13], and eleven in *Leptoglossus occidentalis* Heidemann (Coreidae; phytophagous) [29]. In some insect groups, species with a broad range of hosts were reported to have more chemoreceptors on the mouthparts than those with a more specialized diet [41,42]. We suggest that the sparsity of labial sensilla in flat bugs is associated with their unique mycetophagous feeding habits. Morphological modification of the labium as an adaptation to a particular feeding habit has been observed in other heteropterans [1,3,15]. For example, *Haematoloecha nigrorufa* (Stål) (Hemiptera: Reduviidae), a predatory species specialized on millipedes, possesses hook-shaped last labium, which is believed to facilitate prey catching and manipulation [15]. The labium of *M. yunnana* is constricted distally to form a significantly narrowed tip, a feature rarely observed in other Pentatomomorpha species. Many small comb-shaped cuticular processes (cp) are tightly arranged encircling the opening of the labial apex. Similar structures have been observed in other true bugs, which probably serve to clean the stylets during and after feeding [1,5,13].

**Table 3:** Types of antennal sensilla in Aradidae.

Species	AnCh	AnTr	AnBa	AnCa	AnSt	References
*Aradus betulae* (Linnaeus)	+	+	+	−	+	[19]
*Aradus betulae* (Linnaeus)	+	+	+	−	+	[19]
*Aradus corticalis* (Linnaeus)	+	+	+	−	+	[19,40]
*Aradus corticalis* (Linnaeus)	+	+	+	−	+	[19,40]
*Aradus depressus* (Fabricius)	+	+	+	+	+	[19]
*Brachyrhynchus membranaceus* (Fabricius)	+	+	+	+	+	[19]
*Mezira yunnana* Hsiao	+	+	+	+	+	This study

+, present; −, absent. AnBa, antennal sensilla basiconica; AnCa, antennal sensilla campaniformia; AnCh, antennal sensilla chaetica; AnSt, antennal sensilla styloconica; AnTr, antennal sensilla trichodea.

The most notable feature of aradid mouthparts is the extremely elongated stylet fascicle, which is believed to be an adaptation to their mycetophagous feeding habit [1,19,20,24,39]. According to the latest insights, aradids may use macerate-and-flush or osmotic feeding techniques rather than penetrating individual hyphae, and the elongation of the stylets may be associated with the acquisition of food and reduces the risk of exposure to a certain extent [20]. In natural habitats, mycelium may hide in very narrow crevices that the insect body cannot access. Therefore, the elongated, soft, flexible stylet bundle may have an advantage in obtaining food resources that are difficult to obtain. As we observed, the extremely elongated stylets can allow flat bugs to feed on mycelium in different positions and directions without extensive body movement. Previous studies on Pentatomomorpha species also found that the distally ornamented teeth on mandibular stylets display marked morphological variation across species with different feeding habits. Seed-feeding *P. sibiricus* have prominent and stout teeth which may help in penetrating hard seed coats [13]. The lateral mandibular teeth of the plant-feeding Pentatomidae species tend to be short and blunt, approximately triangular [5], while the sharp, elongated hook-like lateral teeth facilitate immobilization of moving prey [38]. We found that the mandibular apex of *M. yunnana* possesses 8–10 ridge-like and relatively uniform central teeth (ct) and a row of lateral ridges (lr), which may be associated with their unique fungi hosts having a relatively soft surface texture. Combined with the observed feeding processes of *M. yunnana*, we considered that ridge-like teeth are conducive to scratching and penetrating the host surface. The relatively uniform shape of these teeth may facilitate without great resistance the passage of the mandibular stylets through the mycelium composed of entangled hyphae. These may be beneficial to the flat bug to control the direction of the movement of stylets. The inner surface of the mandible has a longitudinal groove that matches the process of the external surface of maxillary stylets and a series of longitudinally arranged small spikes (ss) that have been suggested to increase friction with the external surface of maxillae. Barbs on the inner maxillary surface were documented in many predatory heteropterans, probably serving to filter and triturate large-sized substrates for further digestion and absorption [1,5]. We did not find a similar structure in the mycetophagous *M. yunnana.* Cobben [1] suggested that maxillary barbs are unlikely to assist phytophagous Heteroptera species in extracting sap from host tissue, and loss of this structure was apparently necessary for the evolution of mycetophagous feeding habits in Aradidae. Main features of mouthparts of Aradidae are summarized (Table 4).

## 5. Conclusions

Our study provided detailed description of antennal and mouthparts morphology of *M. yunnana*. The labial tip of *M. yunnana* is constricted. The stylet fascicle is extremely long, and the external surface of the mandible has ridge-like teeth, which differ from other pentatomomorphans feeding on different food types and may help in penetrating the host surface and passing through the mycelium. Combined with the observation of the feeding processes of this species, we suggest that these characteristics reflect a unique adaptation to the mycetophagous feeding habits.

## Figures and Tables

**Table 1:** Measurements of *Mezira yunnana* (mean ± SE).

Sex	Position	Length (µm)	N
Female	Sc	404.01 ± 13.13	3
	Pe	492.61 ± 38.94	3
	Fl I	583.53 ± 26.03	3
	Fl II	528.05 ± 37.01	3
	Sf	13,174.05 ± 605.21	3
	Bo	7104.79 ± 516.78	3
Male	Sc	414.66 ± 2.53	3
	Pe	488.44 ± 15.27	3
	Fl I	587.59 ± 5.53	3
	Fl II	529.39 ± 3.76	3
	Sf	12,371.34 ± 272.23	3
	Bo	6916.45 ± 169.14	3

N = sample number. Bo, body; Fl I, first segment of flagellum; Fl II, second segment of flagellum; Pe, pedical; Sc, scape; Sf, stylet fascicle.

**Table 2:** Morphometric data of antennal and labial sensilla in *Mezira yunnana* (mean ± SE).

Type	Distribution	Length (µm)	Basal Diameter (µm)	N
AnTr I	Fl II	51.71 ± 4.73	3.75 ± 0.29	8
AnTr II	Fl II	40.74 ± 3.90	2.59 ± 0.22	20
AnTr III	Fl II, Fl I, Pe	47.45 ± 2.89	4.12 ± 0.31	20
AnBa I	Fl II	15.51 ± 1.43	2.38 ± 0.17	8
AnBa II	Fl II	8.22 ± 0.15	1.7 ± 0.08	4
AnBa III	Sc	12.73 ± 1.04	2.65 ± 0.31	2
AnCh I	Fl II	74.95 ± 0.42	4.57 ± 0.42	8
AnCh II	Fl I, Sc, Pd	12.07 ± 0.43	1.74 ± 0.10	5
AnCa	Sc	/	/	2
AnSt	all antennal segments	56.26 ± 4.75	3.88 ± 0.59	20
LaTr I	Lb1	34.91 ± 4.65	1.73 ± 0.17	4
LaTr II	all labial segments	16.93 ± 3.72	1.77 ± 0.28	20
LaTr III	Lb3	71.96 ± 4.93	3.54 ± 0.33	10
LaBa I	Lb2	10.14 ± 1.30	2.05 ± 0.44	8
LaBa II	Lb2, Lb3	5.13 ± 0.45	0.80 ± 0.062	4
LaBa III	Lb3	11.35 ± 0.31	1.31 ± 0.082	8
LaCa	Lb3	/	/	/

N = sample number. AnBa I-III, antennal sensilla basiconica I-III; AnCa, antennal sensilla campaniformia; AnCh I-III, antennal sensilla chaetica I-III; AnSt: antennal sensilla styloconica; AnTr I-III, antennal sensilla trichodea I–III; Fl II, second segment of flagellum; La I, first segment of labium; La II, second segment of labium; La III, third segment of labium; LaBa I–III, labial sensilla basiconica I–III; LaCa, labial campaniformia; Sc, scape; LaTr I–III, labial sensilla trichodea I–III; Lm, labrum; Pe, pedical; Fl I, first segment of flagellum.

**Table 4:** Morphological characteristics of mouthparts and diet type of Aradidae species.

Species	Food Notes	Number of Labial Segments	Shape of Last Labial Segment	Types of Sensilla of Labial Tip	Distal Mandibular Stylet; Serration	References
*Aradus betulae* (Linnaeus)	bracket fungi	4	cylindrical	four types of sensilla	slightly expanded with subparallel scales and serrate posterior margins	[20]
*Aradus* sp.	/	/	/	/	more than 20 regular, transverse, parallel	[1]
*Carventus brachypterus* Kormilev	/	3	cone-shaped	/	/	[20]
*Dysodius* sp.	/	/	/	/	grooves	[1]
*Isodermus planus* Erichon	/	4	apex constricted and cleft	sensilla placoid, sensilla setiform	barely expanded with obliquely longitudinal linear ridges and transverse ridges	[20]
*Mezira yunnana* Hsiao	oyster mushroom	4	cone-shaped and constricted distally and cleft	sensilla campaniformia, sensilla basiconica	8–10 central ridge-like teeth and a row of lateral ridges	This study

## Data Availability

All data generated or analyzed during this study are included in this published article.
